# MiR-377 promotes white adipose tissue inflammation and decreases insulin sensitivity in obesity via suppression of sirtuin-1 (SIRT1)

**DOI:** 10.18632/oncotarget.19742

**Published:** 2017-07-31

**Authors:** Jie Peng, Yinghui Wu, Zhao Deng, Yuanfei Zhou, Tongxing Song, Yang Yang, Xiaming Zhang, Tao Xu, Mao Xia, Anle Cai, Zuhong Liu, Jian Peng

**Affiliations:** ^1^ Department of Animal Nutrition and Feed Science, College of Animal Science and Technology, Huazhong Agricultural University, Wuhan 430070, P. R. China; ^2^ The Cooperative Innovation Center for Sustainable Pig Production, Wuhan 430070, P. R. China

**Keywords:** miR-377, SIRT1, obesity, inflammation, insulin-resistance

## Abstract

Obesity is associated with a wide range of metabolic disorders including inflammation and insulin-resistance. Sirtuin-1 (SIRT1) is an important regulator of metabolic homeostasis and stress response pathways in white adipose tissue. However, involvement of microRNAs (miRNAs) in regulating SIRT1 during obesity-induced inflammation and insulin-resistance remains unclear. Here, we found that miR-377 was upregulated in adipose tissue and showed a negative correlation with SIRT1 in chronic high fat diet (HFD)-fed mice. MiR-377 belongs to a large miRNA cluster and functions as an important tumor suppressor in several human malignancies. Recently, it has also gained considerable attention in oxidative stress and diabetic nephropathy. In our present study, we found that overexpression of miR-377 decreased SIRT1 protein abundance and caused inflammation and insulin-resistance in differentiated 3T3-L1 cells. Conversely, miR-377 inhibition increased SIRT1 mRNA and protein levels, ameliorated inflammation and improved insulin sensitivity. Furthermore, we demonstrated that miR-377 targets the 3′-UTR of SIRT1 mRNA directly, and downregulates SIRT1 protein abundance. Inhibition of SIRT1 by EX527 significantly eliminated the downregulation of the inflammation and insulin-resistance levels induced by the miR-377 inhibitor. Furthermore, SIRT1 deficiency intensified adipose tissue inflammation and insulin-resistance, resulting in hepatic steatosis in chronic-HFD-fed mice. In conclusion, our findings suggest that miR-377 promotes white adipose tissue inflammation and decreases insulin sensitivity in obesity, at least in part, through suppressing SIRT1.

## INTRODUCTION

Obesity is a major global health issue [1]. Low-grade chronic inflammation is an important cause of obesity-induced systemic insulin-resistance [2, 3]. Specifically, large numbers of macrophages and T cells are recruited to obese adipose tissue leading to active inflammation, which is thought to alter adipose tissue function, resulting in metabolic disorders and systemic insulin-resistance [4–6].

MicroRNAs (miRNAs) are highly conserved, small non-coding RNAs that regulate gene expression at the post-transcriptional level [7, 8], and are now recognized as biomarkers, prognostic indicators and regulators of normal and abnormal cellular and physiologic function [9]. It has been reported that the miRNA profile of adipocytes changed during lipogenesis, inflammation and type II diabetes mellitus [10–14]. Several miRNAs, such as miR-132 [15], miR-155 [16], miR-130 [17], miR-145 [18], miR-146b [19], and miR-29 [20] have been indentified in obesity-associated inflammation and insulin-resistance in adipocytes. Specifically, in TNFα-treated adipocytes, miR-146b, miR-130 and miR-155 were upregulated. Overexpression of miR-155 resulted in an increased inflammatory state in adipocytes through downregulation of PPARγ [16]. In HFD-induced obese mice, miR-146b knockdown ameliorated insulin-resistance [8]. These results suggest that miRNAs play important roles in regulating adipocyte inflammation and insulin-resistance.

Sirtuin-1 (SIRT1) is an NAD^+^-dependent histone or non-histone deacetylase [21, 22] and plays a central role in regulating obesity-related inflammation and metabolic disease in adipocytes [23–25]. Our previous studies also showed that SIRT1 is an important regulator of adipocyte differentiation and adipogenesis [26–28]. Although the results indicated that SIRT1 was downregulated by HFD in adipose tissue [29, 30], the mechanism was still largely unknown. Many miRNAs have been reported to regulate SIRT1 under different circumstances [31]; however, the mechanism by which miRNAs regulate SIRT1 during obesity-induced inflammation remains unclear.

In the present study, we screened 18 candidate inflammation-related miRNAs targeting SIRT1 and demonstrated that miR-377 decreased SIRT1 protein by direct targeting of the 3′-UTR of SIRT1 mRNA. Furthermore, we investigated the potential mechanism by which miR-377 mediated regulation of SIRT1 expression in adipocyte inflammation and insulin-resistance during obesity. We identified miR-377 as a novel pro-inflammatory miRNA that functions by decreasing SIRT1 protein levels in adipocytes. The miR-377/SIRT1 pathway is implicated as a potential target for attenuating the inflammatory state in adipocytes during obesity.

## RESULTS

### MiR-377 targets the SIRT1 3′-UTR and downregulates SIRT1 at the translational level

**Figure 1:**
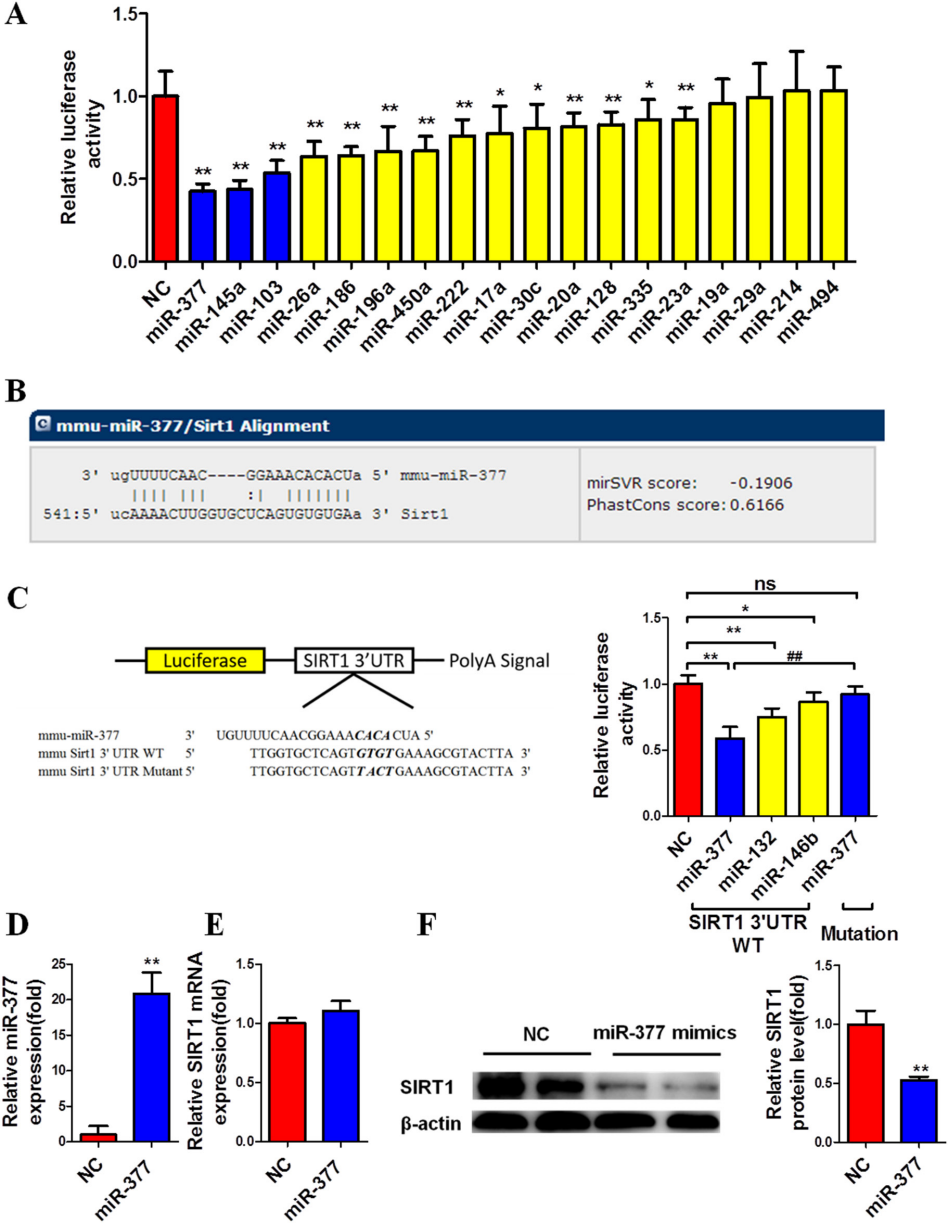
MiR-377 targets the SIRT1 3′-UTR and downregulates SIRT1 protein abundance at the translational level **(A)** Relative luciferase activity of the SIRT1 3′-UTR in BHK cells after co-transfection with negative control (NC) or 18 selected potential miRNAs. *P < 0.05, **P < 0.01 vs. NC (n = 5). **(B)** The potential target sites were predicted by MICRORNA (http: //www. Microrna org/microrna/home.do). **(C)** Relative luciferase activity of wild-type or mutant 3′-UTR after co-transfection with miR-377, miR-132 or miR-146b mimics. *P < 0.05, **P < 0.01 vs. NC; ##*P <* 0.01 vs. miR-377 SIRT1 3’UTR WT; ns, not significant (n = 5). **(D–F)** Mature 3T3-L1 cells were transfected with 100 nM miR-377 mimics for 24 h and then harvested for real-time PCR and immunoblotting analyses. **P < 0.01 vs. NC (n = 3). **(D)** MiR-377 expression. **(E)** SIRT1 mRNA levels. **(F)** SIRT1 protein levels.

To screen and identify the significantly upregulated miRNAs that potentially target the SIRT1 3′-UTR in adipocytes, we searched the miRNA target-prediction programs microRNA.org, miRWalk and TargetScan, and Combined with the sequencing results mentioned above to obtain the potential miRNAs. The 18 selected miRNAs were then evaluated using the SIRT1 3′-UTR pmiR-Glo luciferase reporter vector. Among the miRNAs identified, overexpression of miR-377, miR-145a and miR-103 significantly repressed the activity of luciferase by 50%, with miR-377 showing the most significant effect (Figure 1A).

Searches of microRNA.org conducted to investigate the ability of miR-377 to target the SIRT1 3′-UTR directly revealed a potential binding site (Figure 1B). To evaluate the involvement of this binding site in the inhibitory effect of miR-377, we then generated a mutant 3′-UTR reporter plasmid with four mismatched bases in the seed complementary region. Overexpression of miR-377 had no effect on the luciferase activity of the mutant reporter (Figure 1C); miR-132 and miR-146b, which have been demonstrated to target the 3′-UTR of SIRT1 were used as positive controls [8, 15].

To confirm the impact of miR-377 on SIRT1, differentiated 3T3-L1 cells were transfected with miR-377 mimics for 24 h, then harvested for real-time PCR and immunoblotting analyses. Significant overexpression of miR-377 was confirmed (Figure 1D) although no change in SIRT1 mRNA expression was observed (Figure 1E), while SIRT1 protein levels were markedly downregulated (Figure 1F). These data suggested that miR-377 downregulates SIRT1 through suppression at the translational level. We also found that overexpression of miR-145 and miR-103 did not inhibit SIRT1 translation in differentiated 3T3-L1 cells, although luciferase activity was significantly repressed (Supplementary Figure 1).

### MiR-377 is increased and negatively correlated with SIRT1 during inflammation and HFD-induced obesity

**Figure 2:**
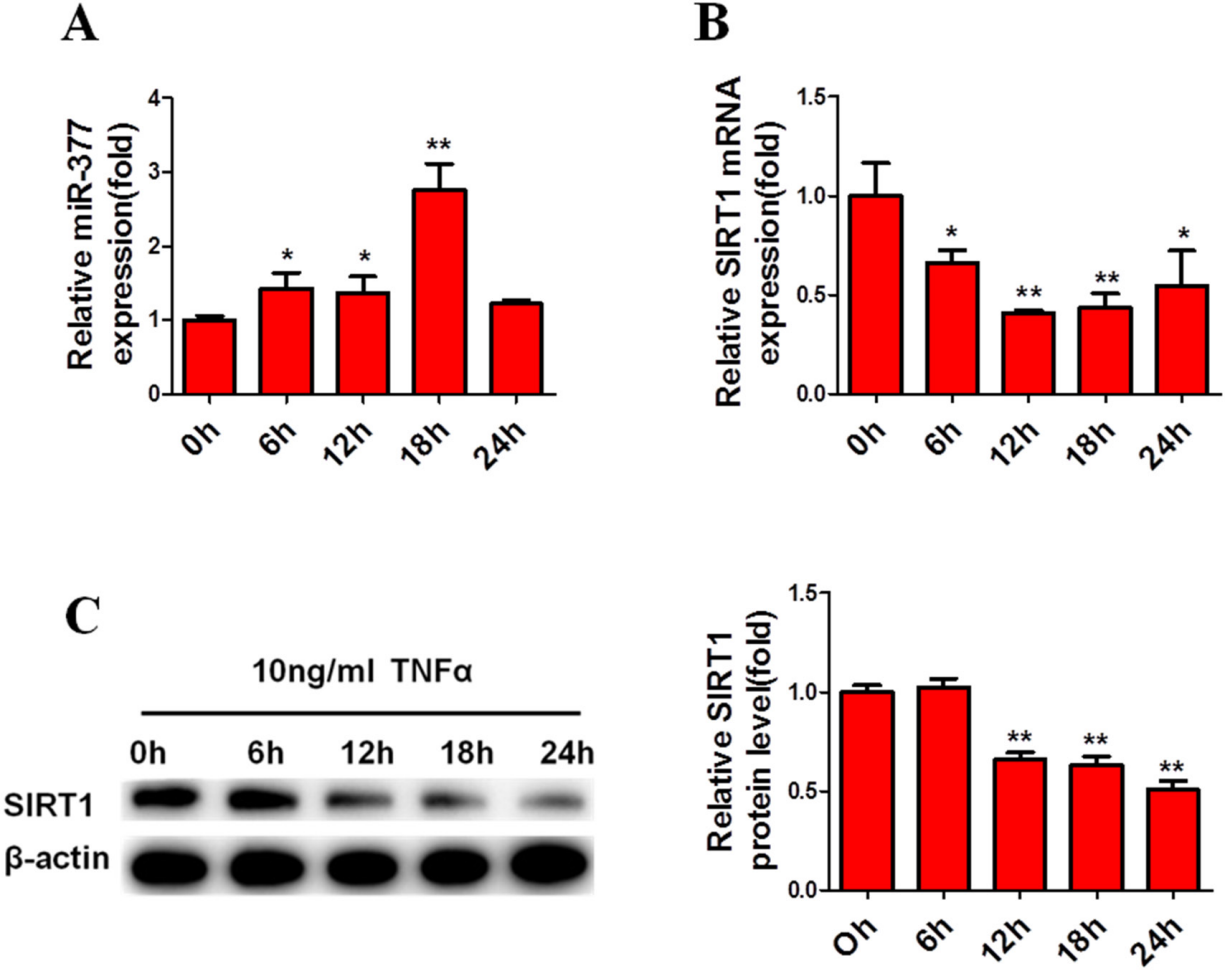
MiR-377 is increased and negatively correlated with SIRT1 under conditions of inflammation Mature 3T3-L1 cells were serum-starved overnight before treatment with 10 ng/ml TNFα. **(A)** MiR-377 expression. **(B)** SIRT1 mRNA levels. **(C)** SIRT1 protein levels. *P < 0.05, **P < 0.01 vs. 0 h (n = 3).

**Figure 3:**
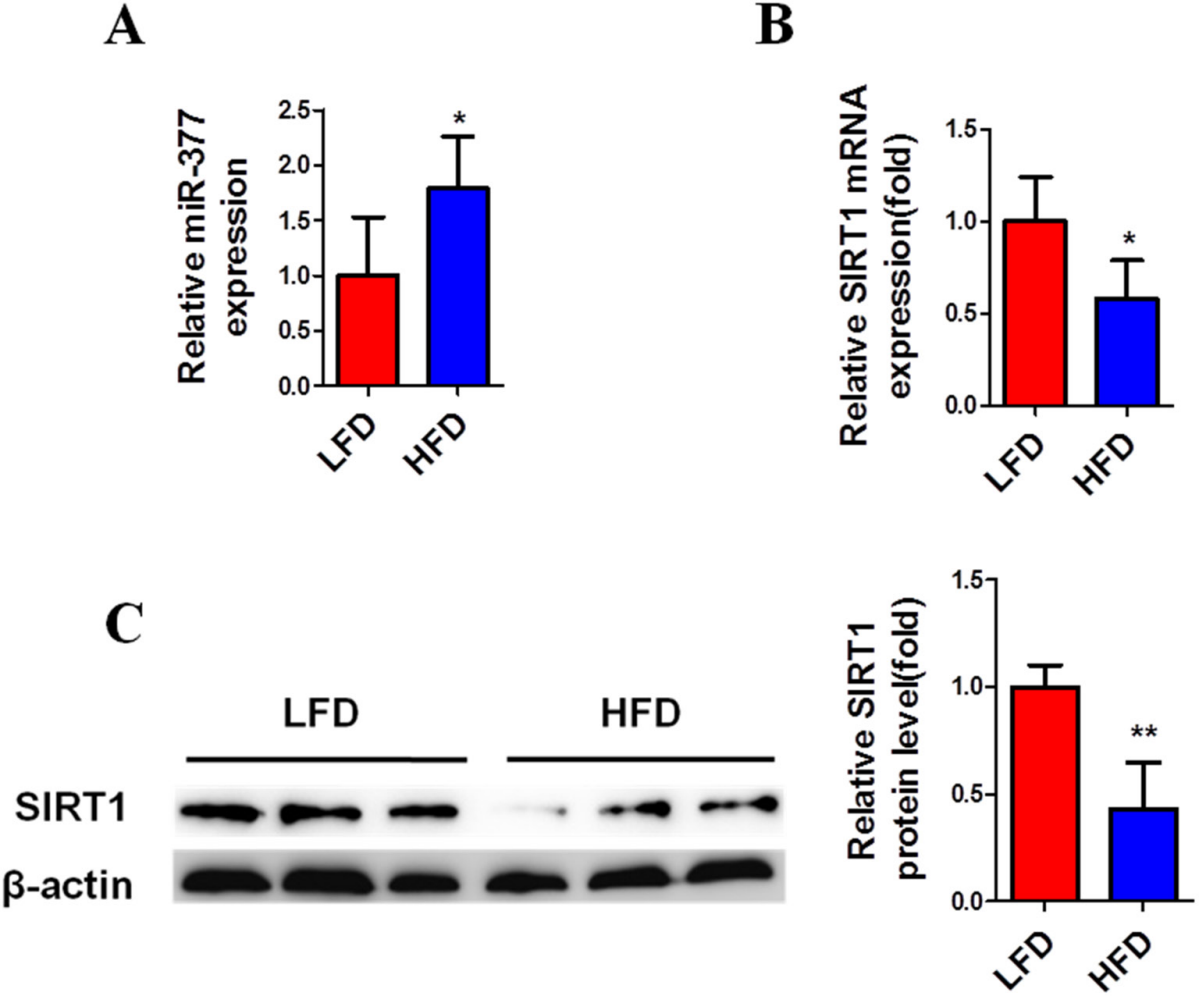
MiR-377 is upregulated by HFD in adipose tissue and negatively correlated with SIRT1 Epididymal fat tissue was collected from wild-type NC or HFD-fed mice, and then harvested for real-time PCR (n = 6–8) and immunoblotting analyses (n = 3). **(A)** MiR-377 expression. **(B)** SIRT1 mRNA levels. **(C)** SIRT1 protein levels. *P < 0.05, **P < 0.01 vs. LFD.

Previous findings have demonstrated that large numbers of macrophages and T cells are recruited to obese adipose tissue leading to active inflammation, which is thought to alter adipose tissue function, resulting in metabolic disorders and systemic insulin-resistance [4–6]. To simulate this inflammatory state, mature 3T3-L1 cells were exposed to 10 ng/ml TNFα [29, 30, 32]. Subsequently, real-time PCR analysis showed that miR-377 expression in mature 3T3-L1 cells was significantly increased after exposure to TNFα for 6 h (Figure 2A). In contrast, SIRT1 mRNA and protein levels were markedly reduced within 24 h (Figure 2B and 2C). Similar changes in miR-377 expression were observed in the epididymis adipose tissue of HFD-fed mice (Figure 3A), while SIRT1 mRNA and protein expression were both decreased (Figure 3B and 3C). Taken together, these results demonstrated that miR-377 expression is increased under conditions of inflammation and HFD-induced obesity in an effect that is negatively correlated with SIRT1 expression.

### MiR-377 promotes inflammation and insulin-resistance in mature 3T3-L1 cells

**Figure 4:**
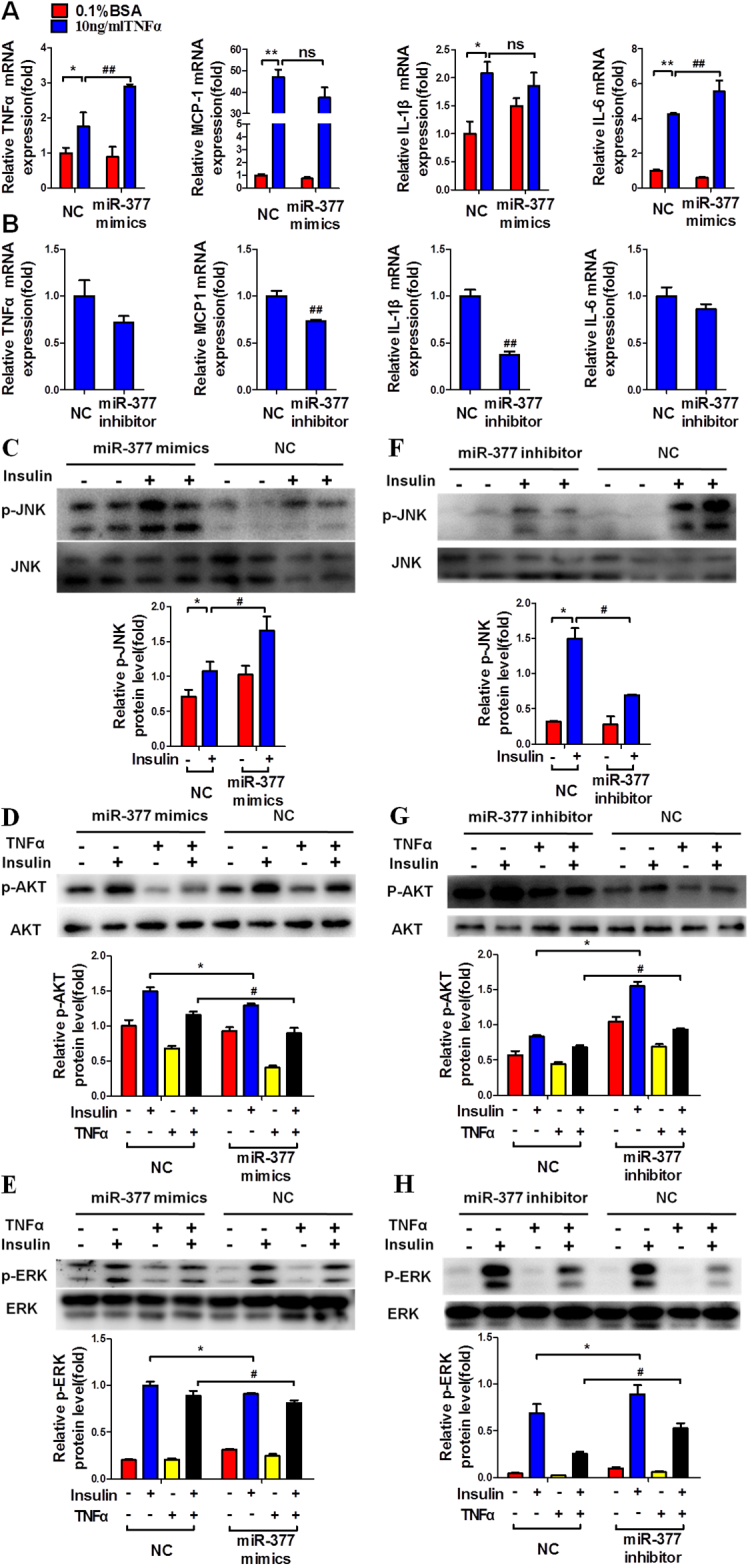
MiR-377 promotes inflammation and insulin-resistance in mature 3T3-L1 cells After transfection with 100 nM miR-377 mimics or inhibitor for 24 h, differentiated 3T3-L1 adipocytes were treated with 10 ng/ml TNFα for 24 h and then stimulated with 100 nM insulin for 15 min. Cells were then harvested for real-time PCR and immunoblotting analyses. **(A)** The effect of miR-377 overexpression on inflammation-related gene expression. *P < 0.05, **P < 0.01 vs. NC 0.1% BSA; ##*P <* 0.01 vs. NC 10 ng/ml TNFα; ns, not significant (n = 3). **(B)** The effect of miR-377 inhibition on inflammation-related gene expression under conditions of TNFα-induced insulin-resistance. ##*P <* 0.01 vs. NC 10 ng/ml TNFα(n = 3). **(C)** The effect of miR-377 overexpression on JNK phosphorylation under conditions of TNFα-induced insulin-resistance. *P < 0.05 vs. NC without insulin; #*P <* 0.05 vs. NC with insulin (n = 3). **(D** and **E)** The effect of miR-377 overexpression on AKT and ERK phosphorylation. *P < 0.05 vs. NC with 0.1% BSA and insulin; #*P <* 0.05 vs. NC with 10 ng/ml TNFα and insulin (n = 3). **(F)** The effect of miR-377 inhibition on JNK phosphorylation under conditions of TNFα-induced insulin-resistance. *P < 0.05 vs. NC without insulin; #*P <* 0.05 vs. NC with insulin (n = 3). **(G** and **H)** The effect of miR-377 inhibition on AKT and ERK phosphorylation. *P < 0.05 vs. NC with 0.1% BSA and insulin; #*P <* 0.05 vs. NC with 10 ng/ml TNFα and insulin (n = 3).

To confirm the role of miR-377 in adipocyte inflammation and insulin sensitivity, we transfected differentiated 3T3-L1 adipocytes with miR-377 mimics or inhibitor for 24 h followed by treatment with TNFα for a further 24 h. MiR-377 overexpression significantly increased the expression of TNFα and IL-6 mRNAs under conditions of TNFα stimulation, while there were no significant differences in the levels of IL-1β and MCP-1 mRNAs (Figure 4A). In contrast, treatment with a miR-377 inhibitor markedly decreased mRNA levels of the inflammatory factors MCP-1 and IL-1β, (Figure 4B). In addition, miR-377 overexpression significantly increased insulin-stimulated JNK phosphorylation (Figure 4C), while AKT and ERK phosphorylation was decreased (Figure 4D and 4E). Conversely, treatment with a miR-377 inhibitor markedly decreased JNK phosphorylation (Figure 4F) and significantly increased the insulin-stimulated AKT (Figure 4G) and ERK (Figure 4H) phosphorylation. These results demonstrated that miR-377 promotes the expression of inflammation-related genes and impairs insulin signaling in TNFα-stimulated mature 3T3-L1 cells.

### MiR-377 promotes inflammation and insulin-resistance via SIRT1 suppression

**Figure 5:**
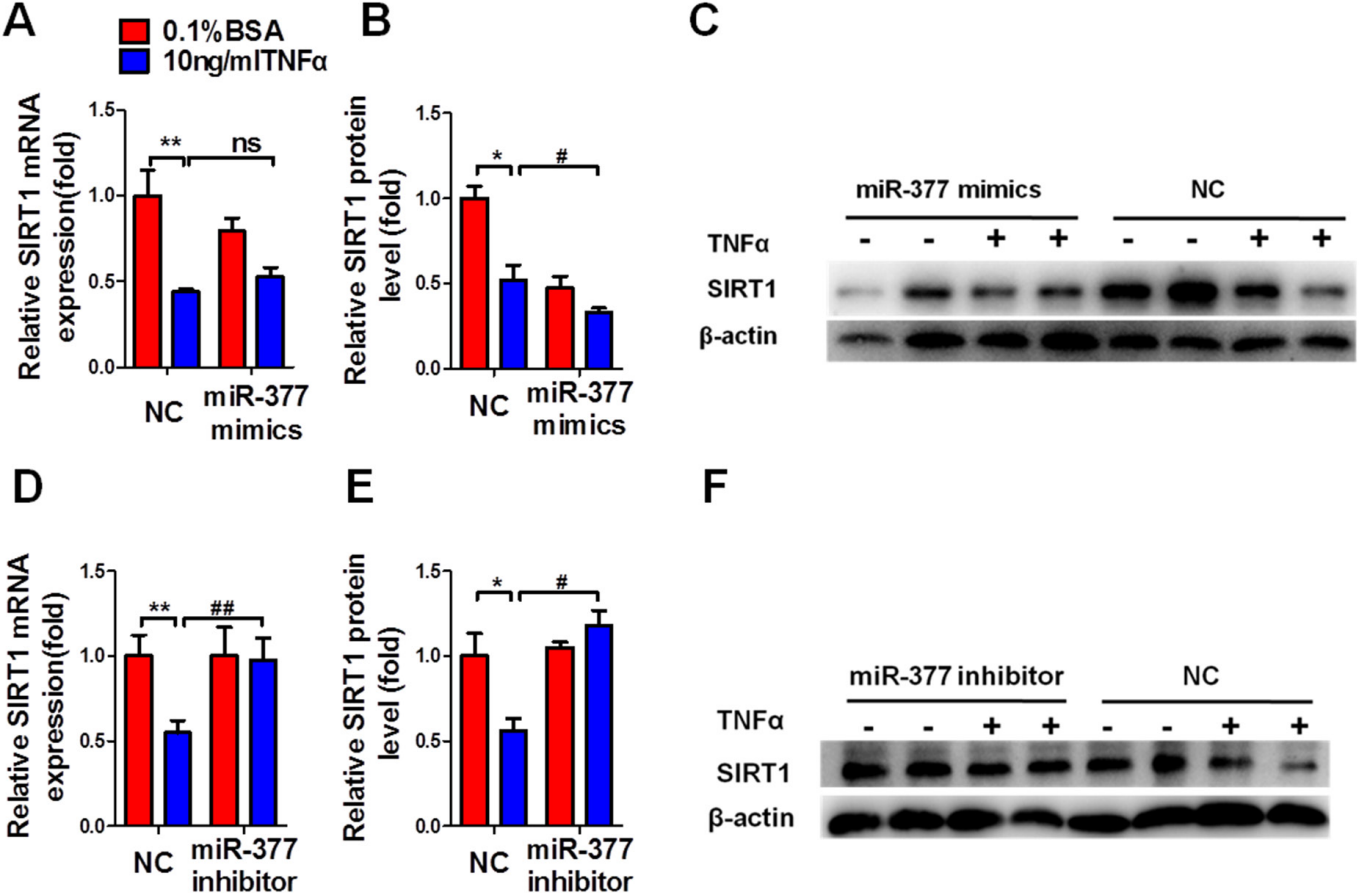
MiR-377 promotes inflammation and insulin-resistance via SIRT1 suppression The effect of miR-377 overexpression on the expression of SIRT1 mRNA **(A)** or protein **(B** and **C)**. The effect of miR-377 inhibition on expression of SIRT1 mRNA **(D)** or protein **(E** and **F)**. *P < 0.05, **P < 0.01 vs. NC 0.1% BSA; #*P <* 0.05, ##*P <* 0.01 vs. NC 10 ng/ml TNFα; ns, not significant (n = 3).

To determine the involvement of miR-377 in SIRT1-mediated regulation of inflammation and insulin sensitivity, we measured the levels of SIRT1 mRNA and protein after transfection with the miR-377 mimics or inhibitor for 48 h. MiR-377 overexpression did not alter SIRT1 mRNA levels (Figure 5A), while SIRT1 protein levels were significantly downregulated regardless of TNFα treatment (Figure 5B and 5C). Conversely, miR-377 inhibition significantly increased SIRT1 mRNA levels (Figure 5D) and protein abundance (Figure 5E and 5F) under conditions of TNFα stimulation.

### SIRT1 inhibition is required for the miR-377-mediated inflammation and insulin-resistance

**Figure 6:**
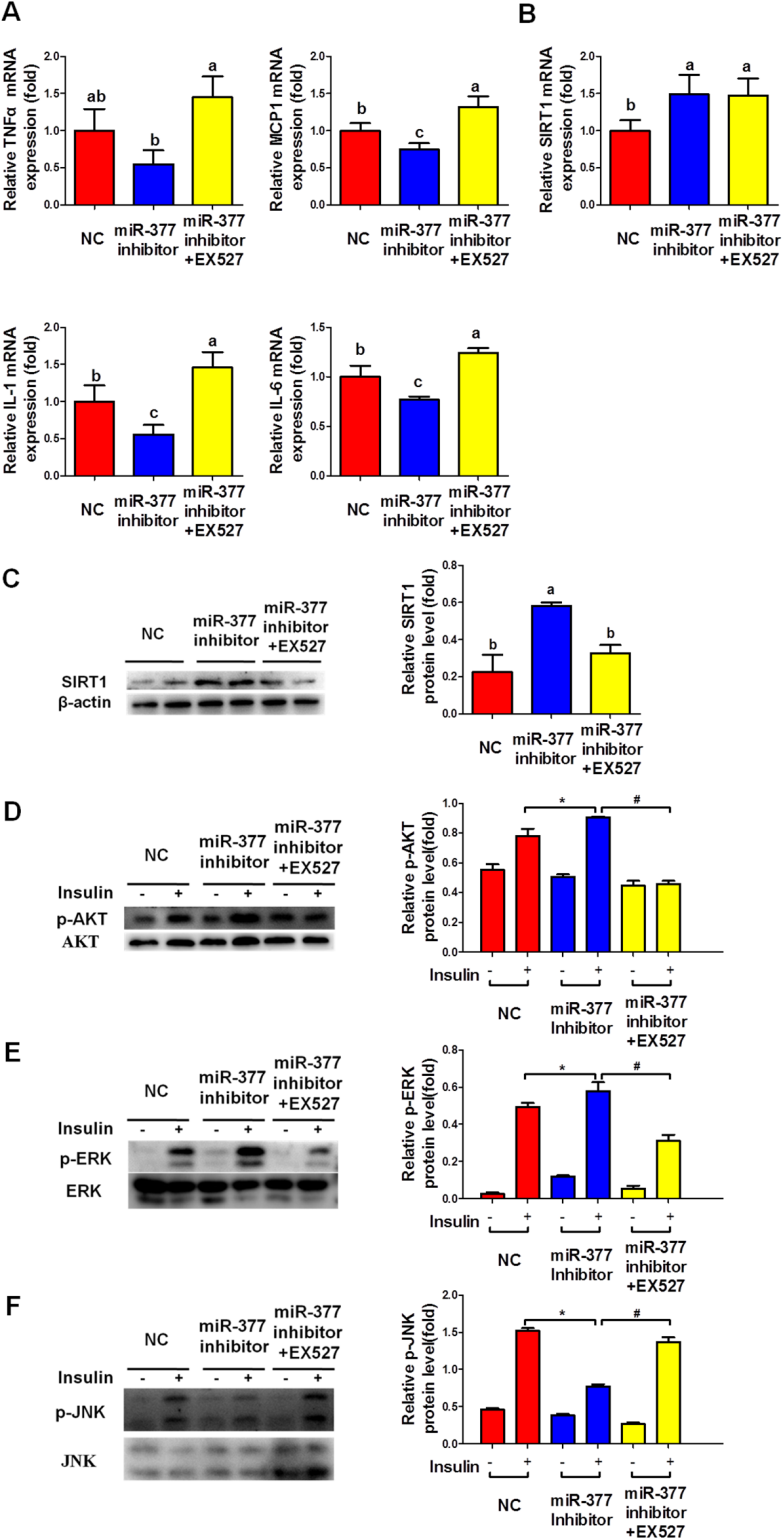
SIRT1 inhibition is required for the miR-377-mediated inflammation and insulin-resistance After transfection with 100 nM miR-377 inhibitor for 24 h, differentiated 3T3-L1 adipocytes were treated with 10 ng/ml TNFα and 10 μM EX527 for 24 h and then stimulated with 100 nM insulin for 15 min. Cells were then harvested for real-time-PCR and immunoblotting analyses. **(A)** Expression of inflammation-related genes. Different letters denote significant differences, *P* < 0.05 (n=3). **(B** and **C)** SIRT1 mRNA and protein levels. Different letters denote significant differences, *P* < 0.05 (n=3). **(D-F)** p-AKT, p-ERK and p-JNK protein levels. *P < 0.05 vs. NC with insulin; #*P <* 0.05 vs. miR-377 inhibitor with insulin (n = 3).

To further confirm the involvement of the miR-377/SIRT1 pathway in the regulation of adipocyte inflammation and insulin sensitivity, we transfected differentiated 3T3-L1 cells with miR-377 inhibitor for 24 h prior to treatment with EX527, a selective SIRT1 inhibitor, and TNFα for a further 24 h. MiR-377 inhibition markedly decreased mRNA levels of the inflammatory factors MCP-1, IL-6 and IL-1β (Figure 6A), while SIRT1 expression was upregulated at both the mRNA (Figure 6B) and protein levels (Figure 6C). Furthermore, EX527 significantly eliminated the downregulation of the inflammatory factors (Figure 6A) and the phosphorylation of JNK (Figure 6D) induced by the miR-377 inhibitor alone. EX527 also diminished the upregulation of the insulin-stimulated AKT and ERK phosphorylation observed following miR-377 inhibition alone (Figure 6E and 6F). Taken together, these results suggested that SIRT1 inhibition is required for miR-377-mediated inflammation and insulin-resistance.

### SIRT1 deficiency intensifies adipose tissue inflammation, insulin-resistance and hepatic steatosis in HFD-fed mice

To determine the role of SIRT1 in the regulation of adipose tissue inflammation and insulin sensitivity *in vivo*, SIRT1-deficient mice (whole body) were used to generate an obesity model. Our previous study indicated that different SIRT1 genotypes are associated with different adipogenic phenotypes in adipocytes [28]. To assess this, three types of SIRT1 mice were used in our study. In this model, SIRT1^+/+^, SIRT1^+/-^ and SIRT1^-/-^ mice were fed a 60% HFD for 12 weeks, while SIRT1^+/+^ mice fed a 10% LFD as a negative control. Body composition analysis showed that there were no differences in body weight and liver weight between the HFD groups (Supplementary Figure 2E, 2F and 2L), while the body mass index (BMI) was significantly increased in SIRT1^-/-^ mice (Supplementary Figure 2G). The weight and percentage of white adipose tissue (WAT) and brown adipose tissue (BAT) were increased in SIRT1^+/-^ mice (Supplementary Figure 2H–2K), while, SIRT1^-/-^ mice exhibited a 20% reduction in WAT and a 58% reduction in BAT. Oil red-O staining revealed that SIRT1-deficiency also resulted in hepatic steatosis (Supplementary Figure 2A).

As expected, intake of HFD induced hyperglycemia and glucose intolerance (Supplementary Figure 3A and 3E) in both SIRT1^+/-^ and SIRT1^-/-^ mice as demonstrated by the significantly increased area under the curve of IPGTT compared with that in WT mice (Supplementary Figure 3C); however, there were no differences in IPITT among the HFD groups (Supplementary Figure 3B and 3D), indicating that a higher dose of insulin of 1 IU/kg [30] or 1.2 IU/kg body weight [33] might be needed. We also found that the mRNA expression of the macrophage marker genes, F4/80 and CD11, were markedly increased in epididymis adipose tissue from HFD SIRT1^+/-^ and SIRT1^-/-^ mice (Supplementary Figure 3F and 3G). In accordance with these results, plasma levels of TNFα, IL-1β and IL-6 were increased (Supplementary Figure 3H, 3I and 3J). These results demonstrated that SIRT1 is an important coordinator in the regulation of metabolic homeostasis, including processes such as inflammation, insulin-resistance and hepatic steatosis in HFD-induced obesity.

## DISCUSSION

Many studies have been conducted to investigate the function of miR-377, which belongs to a large miRNA cluster on distal mouse chromosome 12 and human chromosome 14 [33, 34]. MiR-377 functions as a tumor suppressor in several human malignancies, including malignant melanoma [35], clear cell renal cell carcinoma [36], and non-small-cell lung cancer [37]. Tsirimonaki et al. [38] reported that miR-377 expression is regulated by protein kinase C-ε (PKC-ε) in human nucleus pulposus cells. A rencent study showed that the long non-coding RNA, NEAT1, promotes non-small cell lung cancer progression via regulation of the miR-377/E2F3 pathway [39].

Recently, miR-377 has gained considerable attention with regard to its involvement in oxidative stress and diabetic nephropathy. Wang et al. [40] reported that miR-377 was upregulated, leading to increased fibronectin production in diabetic nephropathy. Another study also showed that miR-377 is upregulated in fructose-caused metabolic syndrome, leading to exacerbated podocyte oxidative stress, inflammation and injury [41]. However the potential role of miR-377 in adipocyte inflammation and insulin-resistance remains to be elucidated.

In the present study, we identified miR-377 as a novel pro-inflammatory miRNA that functions by direct targeting of the 3′-UTR of SIRT1 mRNA and decreasing SIRT1 protein levels in adipocytes. Inhibition of SIRT1 by EX527 significantly eliminated the downregulation of the inflammation and insulin-resistance induced by the miR-377 inhibitor. Furthermore, SIRT1-deficiency intensified adipose tissue inflammation and insulin-resistance, resulting in hepatic steatosis in chronic-HFD-fed mice.

A previous study indicated that obesity and inflamation induced the cleavage of SIRT1 protein in adipose tissue and differentiated 3T3-L1 cells by caspase-1 [30]. Notably, our studies revealed that miR-377 inhibition increased SIRT1 transcription and rescued TNFα-induced SIRT1 cleavage. However, we did not observe decreased SIRT1 transcription following miR-377 overexpression for 48 h, indicating the possible existence of negative feedback regulation.

Although some studies have indicated the beneficial effects of SIRT1 activation on glucose homeostasis, this issue is still open to debate. It was recently reported that insulin sensitivity was enhanced by inhibition of SIRT1 by RNAi or histone deacetylase inhibitor treatment [42]. Erion et al. also indicated that SIRT1 knockdown in liver decreased basal hepatic glucose production and increased hepatic insulin responsiveness in diabetic rats [43]. In addition, adipocyte-specific SIRT1 knockout promoted PPAPγ activity and insulin sensitivity under chronic-HFD conditions and obesity [44]. In accordance with these results, although we found that miR-377 overexpression significantly downregulated SIRT1 protein levels in mature 3T3-L1 cells, we did not observe a marked inflammatory effect, despite significant upregulation of TNFα, IL-6 and p-JNK (Figure 4A and 4C).

Similarly, in SIRT1 knockout mice, although intake of HFD induced a higher level of glucose intolerance in SIRT1^-/-^ mice than SIRT1^+/-^ mice (Supplementary Figure 3A and 3E), there were no differences in the expression of macrophage marker genes epididymal fat between the two groups (Supplementary Figure 3F and 3G). This partly reflects the fact that SIRT1 deficiency in adipose tissue may not be sufficient to reduce systemic insulin sensitivity. In contrast, Sirt1 has been reported to regulate insulin secretion in β cells [45], indicating that the regulatory role of SIRT1 in other organs may be more important. However, miR-377 inhibition markedly decreased the production of inflammatory factors and improved insulin sensitivity. Furthermore, inhibition of SIRT1 by EX527 significantly eliminated the downregulation of the inflammatory factors induced by the miR-377 inhibitor. These findings demonstrate the importance of SIRT1 in metabolic homeostasis, although the underlying mechanism remains to be clarified.

It should be noted that we did not further confirm the role of miR-377 in the regulation of SIRT1 expression and SIRT1-mediated inflammation and insulin-resistance by using miR-377 mimics and inhibitor in an animal model. In addition, the mechanism by which HFD and TNFα stimulate the expression of miR-377 in adipose requires further investigation.

**Figure 7:**
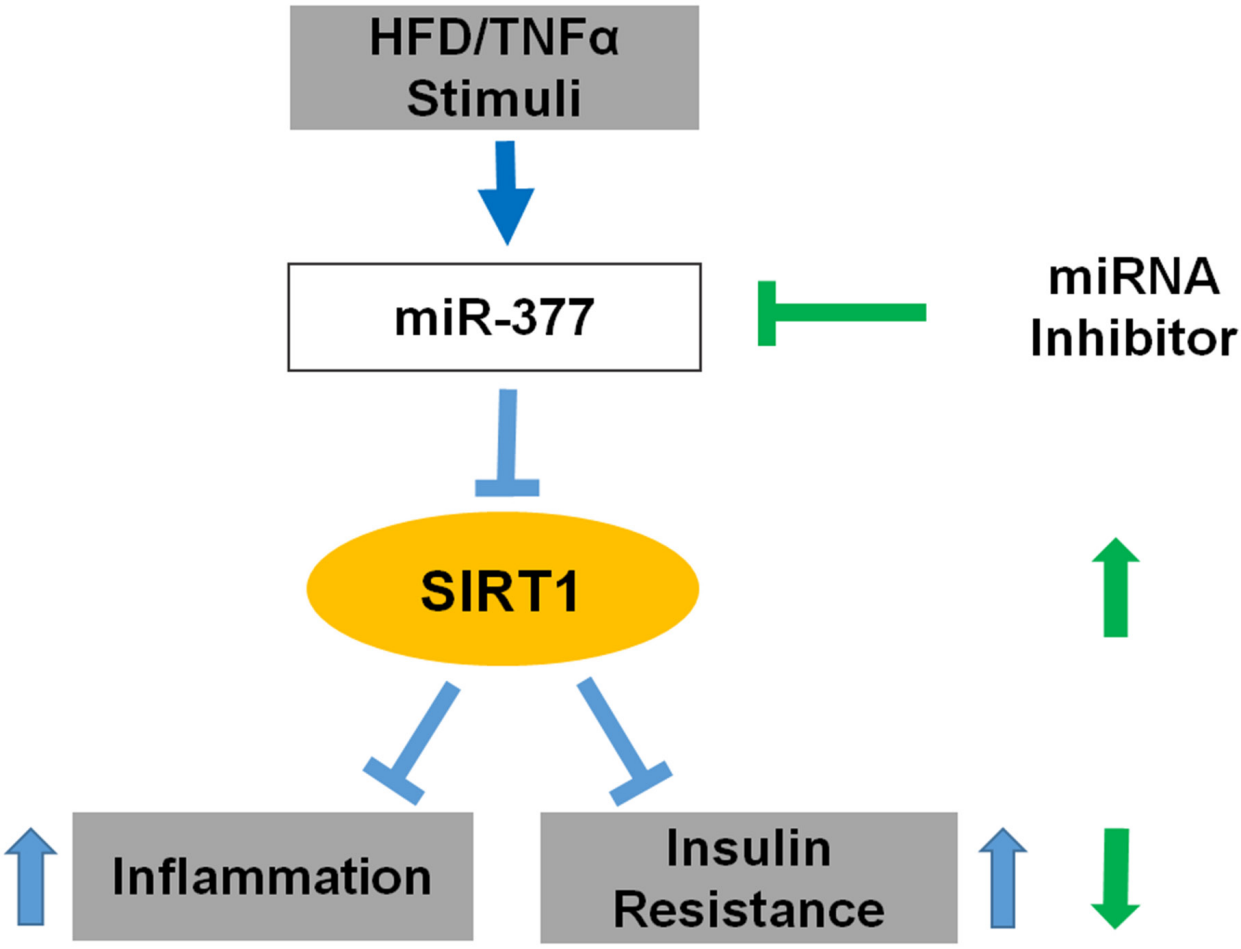
Schematic representation of how miR-377 impairs adipocytes inflammation and insulin-resistance Obesity and inflammation induce upregulation of miR-377, which binds to the SIRT1 3' - UTR and downregulates SIRT1 protein levels. Consequently, inflammation and insulin-resistance are intensified in adipocyte under these conditions. Conversely, inhibition of miR-377 upregulates SIRT1 mRNA levels and protein abundance, ameliorates inflammation and improves insulin sensitivity.

In conclusion, our study demonstrates that miR-377 functions as a novel inhibitor of SIRT1 in obesity-induced inflammation and insulin-resistance. MiR-377 upregulation results in post-transcriptional SIRT1 silencing, which contributes to adipose tissue inflammation and insulin-resistance (Figure 7). Therefore, the miR-377/SIRT1 pathway is implicated as a potential target for attenuating the inflammatory state in adipocytes during obesity.

## MATERIALS AND METHODS

### Animal experiments

C57BL/6J mice were obtained from The Hubei Research Center of Laboratory Animals (China). Male heterozygous knockout (SIRT1^+/-^) mice on the 129/ICR background were gifts from the Institute for Nutritional Sciences, Shanghai Institutes for Biological Sciences, the Chinese Academy of Sciences [46]. SIRT1^+/-^ mice were backcrossed with C57BL/6 mice for five generations to obtain the C57BL/6 gene background. The genotyping for SIRT1 was carried out as described previously [28]. All animal procedures were performed according to the Guidelines of the Institutional Animal Care and Use Committees at the Huazhong Agricultural University. For high fat diet (HFD)-induced obesity studies, mice with different genotypes were systematically assigned to four groups (n = 6–8 mice/group).: (I) SIRT1^+/+^ mice placed on a low fat diet (LFD) (10% kcal fat, Research Diet, D12450, USA); (II) SIRT1^+/+^ mice fed a HFD (60% kcal fat; Research Diet, D12492); (III) SIRT1^+/-^ mice fed a HFD; (IV) SIRT1^-/-^ mice fed a HFD. All mice were allowed free access to food and water. The diet for each group was started at 6 weeks of age and sustained for 12 weeks.

For introperitoneal glucose tolerance tests (IPGTTs) and introperitoneal insulin tolerance tests (IPITTs), 18-week-old mice were fasted 6 h prior to injection with either 2 g/kg glucose or 0.6 IU/kg insulin (Novolin, Denmark), respectively, at 3-day intervals. Blood samples were obtained from the caudal vein at 0, 15, 30, 60, and 120 min for plasma glucose analyses [47, 48]. Three days later, retro-orbital blood samples were obtained and the mice were then sacrificed by cervical dislocation. Blood was immediately centrifuged (4,000 rpm, 10 min, 4°C), and the plasma was separated and stored at -80°C until assayed. The WAT, BAT and liver depots were collected and weighed. Partial tissues samples were fixed separately in 4% paraformaldehyde and embedded in paraffin. Oil red-O and hematoxylin and eosin (H&E) staining (n = 3 mice/group) were conducted by Goodbio Technology, China. The remainde of the samples were rapidly frozen in liquid nitrogen and stored at -80°C for further processing. Plasma TNFα, IL-1β and IL-6 levels were measured (n = 6–8 mice/group) using ELISA kits (Neobioscience, China).

### Cell culture and differentiation

BHK and 3T3-L1 cells (American Type Culture Collection) were cultured in high glucose Dulbecco’s modified Eagle’s medium (DMEM) (Gibco, Grand Island, USA) supplemented with 10% fetal bovine serum (FBS) (Gibco, USA) and 1% penicillin/streptomycin (Mediatech, USA) at 37°C under 5% CO_2_. For adipogenic differentiation, 2-days post-confluent 3T3-L1 cells (designated day 0) were treated with MDI (1 mM dexamethasone, 0.5 mM isobutyl-methylxanthine, 10 mg/ml insulin). After 2 days, the cells were re-fed with medium supplemented with 10% FBS ad containing only 10 mg/ml insulin for 2 days. Cells were then maintained in medium supplemented with 10% FBS for a further 4 days.

### Plasmids and 3' UTR luciferase reporter assays

The 3′-UTR sequence of the SIRT1 gene was generated from a mouse cDNA library and cloned into the pmiR-Glo reporter vector (Invitrogen, USA) at the *Pme*I and *Xho*I sites. The primer sequences were as follows: mSirt1-*Pme*I Forward 5'-AGCTTT**GTTTAAAC**GAAGCT GTCCGGATTCAGGA-3', mSirt1-*Xho*I Reverse 5'-CCG**CTCGAG**TCCAGTCATTAAACGGTCTACA-3'. This wild-type plasmid was designated SIRT1-Wt. The potential target sites were predicted by TargetScan (http://www.targetscan.org/), miRWalk (http://zmf.umm.uni-heidelberg.de/apps/zmf/mirwalk2/) and MICRORNA (http: //www. Microrna.org/microrna/home.do). SOE PCR was used to generate the mutant binding site plasmid [49]. This plasmid was designated SIRT1-Mutation. The mutated primer sequences were as follows: Forward 5'-TTGGTGCTCAGTT**ACTG**AAAGCGTACTTA-3', Reverse 5' TAAGTACG CTTT**CAGT**AACTGAGCACCAA-3'. For luciferase reporter assays, BHK cells seeded in a 48-well plate (1×10^5^ cells/well) were co-transfected with 0.75 pmol (30 nM) of miRNA mimics or negative control (GenePharma, Shanghai, China) and SIRT1-Wt or SIRT1- Mutation using lipofectamine 2000 (Invitrogen, USA). Cell lysates were obtained 24 h after transfection and luciferase activity was analyzed by using the Dual Luciferase Reporter Assay System (Promega, USA).

### Cell treatment and transient transfection

Lipid-based siRNA transfection of adipocytes in suspension was performed as previously described [50]. Briefly, differentiated 3T3-L1 adipocytes were rinsed with PBS and detached from the plate by trypsin (Gibco, USA) treatment for approximately 5 minutes (1 ml of 0.25% trypsin/10 cm^2^ plate). Cells were then resuspended gently in medium supplemented with 10% FBS and collected by centrifugation at 1,000 rpm for three minutes at room temperature. The pelleted adipocytes were resuspended gently in medium supplemented with 10% FBS again, and counted using a hemocytometer after the addition of trypan blue. The adipocytes were re-plated in 12-well plates (8×10^5^ cells/well) in the presence of 100 nM miRNA mimics/inhibitor and 5 μL lipofectamine 2000 and incubated at 37°C under 5% CO_2_ for 24 h. To induce inflammation, transfected cells were treated 10 ng/ml TNFα (Peprotech, USA) or 0.1% bovine serum albumin as a control for a further 24 h prior to stimulation for 15 min with or without 100 nmol/l insulin (Sigma, St. Louis, MO, USA). Cells were then harvested for real-time PCR and immunoblotting analyses. To suppress SIRT1 activity, 10 μM EX-527 (SELLECK, USA) was added for 24 h under TNFα treatment of differentiated 3T3-L1 adipocytes at 24 h after transfection with the miR-377 inhibitor.

### RNA isolation and quantitative real-time PCR

Total RNA from tissues or cultured cells was extracted using the TRIzol reagent (Invitrogen, USA). For mRNA quantification, 2 μg RNA was converted into cDNA using ReverTra Ace qPCR RT Kit (TOYOBO, Japan) according to the manufacturer’s protocol. Quantitative real-time PCR was performed using iQ SYBR green Supermix (BioRad, USA) on a CFX™ 384 Touch qPCR system (BioRad, USA). β-actin served as an endogenous control [8, 28, 51, 52]. The primers used are listed in Supplementary Table 1. For miRNA quantification, stem-loop RT-PCR was performed using the ReverTra Ace qPCR RT Kit (TOYOBO, Japan). In detail, reverse transcription was performed in a reaction mix (20 μl) consisting of 1 μg of purified total RNA, 2 μl of stem-loop RT primers (10 mM) (miR-377:U6, 1:1), 2.5 μl of 5× RT Buffer, 2 μl of 10 mM dNTPs, 1 μl of ReverTra Ace (100 U/μl), 0.5 μl of RNase inhibitor (40 U/μl), and DEPC-H_2_O to 20 μl. The reaction mix was incubated for 60 min at 42°C, 5 min at 95°C, and then held at 4°C. U6 served as an endogenous control. MiR-377 and U6 stem-loop primers are listed in Supplementary Table 1. Melting curves were used to evaluate non-specific amplification. The relative expression level was calculated using the 2^−ΔΔCt^ method.

### Western blotting

Total proteins were extracted from cultured cells and adipose tissue with protein lysis buffer RIPA (Goodbio Technology, China) supplemented with the protease inhibitor PMSF and a phosphatase inhibitor according to the manufacturer’s protocol. Proteins were separated by sodium dodecyl sulfate polyacrylamide gel electrophoresis. The separated proteins were transferred to a polyvinylidene difluoride membrane before immunoblotting. The following primary detection antibodies used in this study: anti-SIRT1 (Cell Signaling Technology CST, No. 2028, USA), anti-phospho-AKT (Ser 307) (CST, No. 12694S, USA), anti-AKT (Affinity Biosciences, AF6261, USA), anti-p-ERK1/2 (Thr 202/Tyr 204) (Affinity Biosciences, AF1015), anti-ERK1/2 (Affinity Biosciences, AF0155), anti-phospho-JNK1/2/3 (Thr183+Tyr185) (Affinity Biosciences, AF3318, USA), anti-JNK (Santa Cruz Biotechnology, sc-7345, USA) and anti-β-actin (Santa Cruz Biotechnology, sc-47778, USA). Anti-mouse or anti-rabbit IgG-HRP (Invitrogen, USA) were used as secondary detection antibodies. Finally, immunoreactive bands were detected using the Super Signal chemiluminescence detection kit (Thermo Scientific, USA) in imaging system FluorChem M apparatus (CareStream 2200 PRO, USA). β-actin used as an endogenous control. The density of the bands was analyzed using image analysis software (IMAGE J).

### Statistical analysis

Data are expressed as the mean±standard deviation (SD) of at least three independent experiments. A two-tailed Student’s *t*-test (SAS statistical package, v 8.2, SAS Inst., Inc., Cary, NC) was used to compare differences between two groups (Figure 1–5 and 6D–6F). The Tukey post-hoc multiple comparison test was performed to compare significant variations (Figure 6A-6C and Supplementary Figure 2 and 3). *P* < 0.05 was considered to indicate statistical significance.

## SUPPLEMENTARY MATERIALS FIGURES AND TABLE


