# Supplementary material for: MiR-377 promotes white adipose tissue inflammation and decreases insulin sensitivity in obesity via suppression of sirtuin-1 (SIRT1)
Source: Oncotarget. 2017 Jul 31;8(41):70550–63. doi: 10.18632/oncotarget.19742 (PMC5642576; doi:10.18632/oncotarget.19742)
Supplement: Supplementary file 1 [file oncotarget-08-70550-s001.pdf]

MiR-377 promotes white adipose tissue inflammation and decreases insulin sensitivity in obesity via suppression of sirtuin-1 (SIRT1)

SUPPLEMENTARY MATERIALS

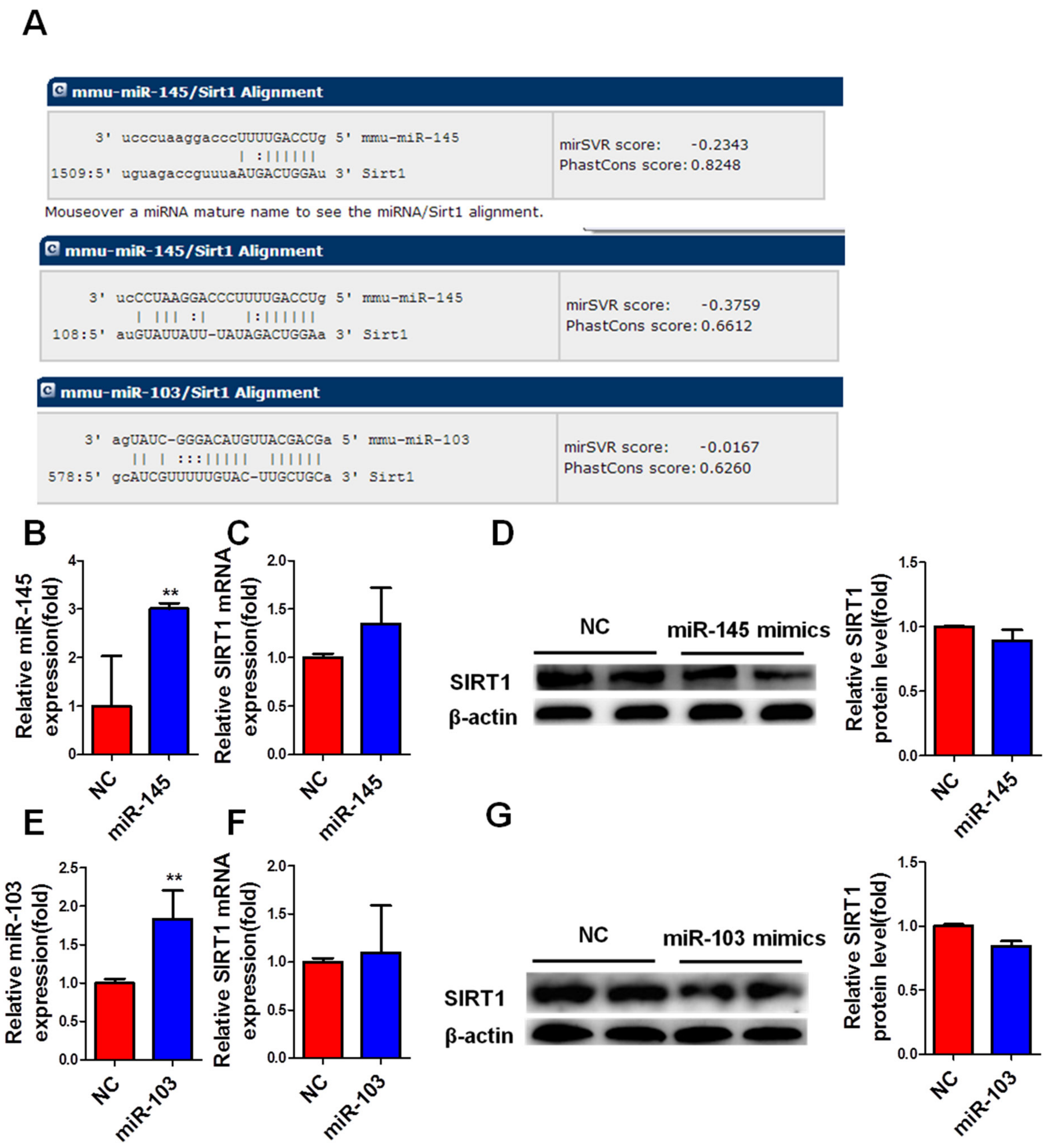

Supplementary Figure 1: Overexpression of miR-145 and miR-103 did not inhibit the translation of SIRT1 in differentiated 3T3-L1 cells. **(A)** The potential target sites were predicted by MICRORNA. **(B–G)** Mature 3T3-L1 cells were transfected with 100 nM miR-145 or miR-103 mimics, respectively, for 24 h and then harvested for real-time PCR and immunoblotting analyses. **(B)** MiR-145 expression. **(C)** SIRT1 mRNA levels. **(D)** SIRT1 protein levels. **(E)** MiR-103 expression. **(F)** SIRT1 mRNA levels. **(G)** SIRT1 protein levels. ***P* < 0.01 vs. NC (n=3).

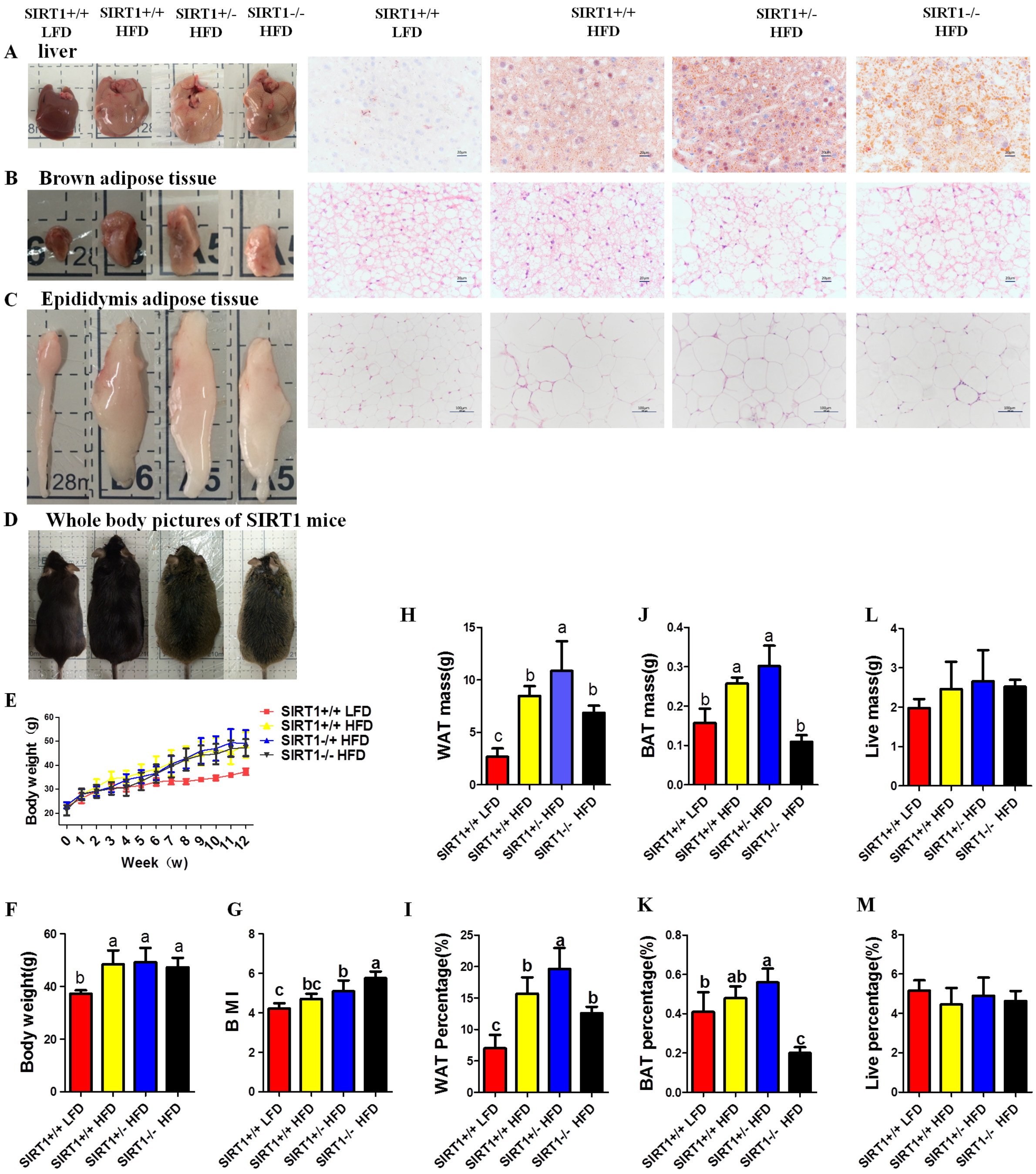

Supplementary Figure 2: SIRT1-deficiency affects adipogenesis and results in hepatic steatosis in HFD-fed mice. Mice with different genotypes received a LFD or a HFD for 12 weeks. **(A)** Livers and oil red-O staining of liver tissue; scale bar, 20 μm (n =3). **(B)** Brown adipose tissue (BAT) and histological (hematoxylin and eosin) staining of sections; scale bar, 20 μm (n =3). **(C)** Epididymal adipose tissue and histological (hematoxylin and eosin) staining of sections; scale bar, 100 μm (n =3). **(D)** Whole body images of SIRT1 mice. **(E)** Body weight during 12 weeks of HFD (n =6-8). **(F)** Body weight at week 12 (n =6-8). **(G)** Body mass index (BMI) (n =6-8). **(H** and **I)** White adipose tissue mass and percentage (n =6-8). **(J** and **K)** BAT mass and percentage. **(L** and **M)** Liver mass and percentage (n =6-8). Different letters denote significant differences, $P < 0.05$.

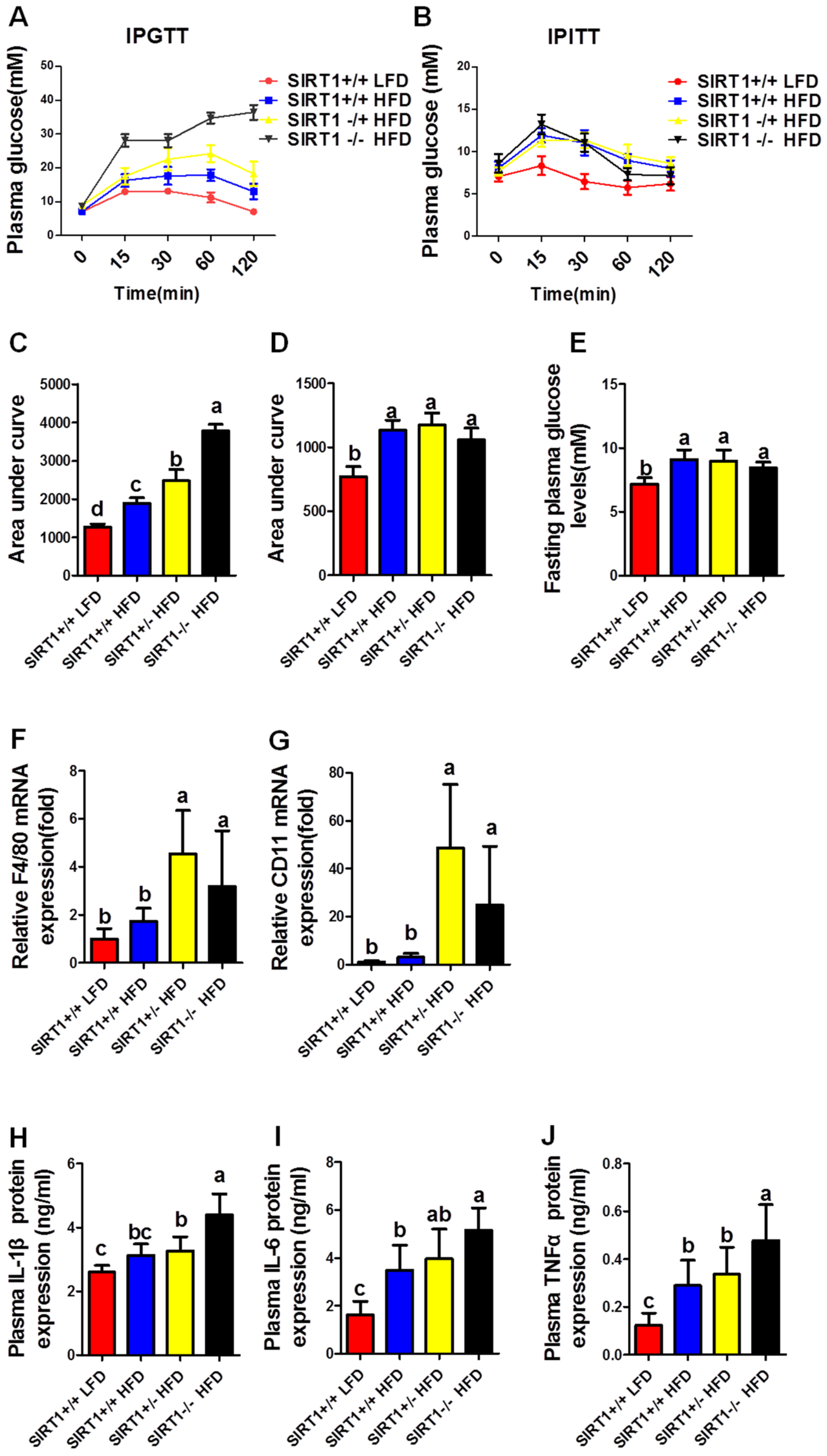

Supplementary Figure 3: SIRT1-deficiency intensifies adipose tissue inflammation and insulin-resistance in HFD-fed mice. Mice with different genotypes received a LFD or a HFD for 12 weeks prior to IPGTT or IPITT evaluation (n = 6–8). **(A)** IPGTT, 2 g/kg glucose. **(B)** IPITT, 0.6 IU/kg insulin. **(C)** Area under the curve from IPGTT. **(D)** Area under the curve from IPITT. **(E)** Fasting plasma glucose. **(F and G)** mRNA levels of macrophage specific antigen F4/80 and CD11 from epididymal adipose tissue. **(H–J)** Serum levels of inflammatory factors IL-1β and IL-6 and TNFα. Different letters denote significant differences, $P < 0.05$.

Supplementary Table 1: The primer used in this study

Gene	Forward Primer	Reverse Primer
F4/80	5' AGGGCAGGGATCTTGGTTATG 3'	5' AGCTGCACTCTGTAAGGACAC 3'
Sirt1	5' CCTGACTTCAGATCAAGAGACGGTA 3'	5' CTGATTAAAAATGTCTCCACGAACAG 3'
CD11	5' TGGATGCAGAGAAGCTGACAC 3'	5' CAACCACCACCCAGGAAC TAT 3'
β-actin	5' GGCACCACACCTTCTACAATG 3'	5' GGGGTGTTGAAGGTCTCAAAC 3'
TNFα	5' CGGAGTCCGGGCAGGTCTACTTT 3'	5' GTCCAGGTCACTGTCCCAGCATC 3'
IL-1β	5' CAAGCAATACCCAAAGAAGAAGA 3'	5' ATTAGAAACAGTCCAGCCCATAC 3'
IL-6	5' CCAGAGATACAAAGAAATGATGG 3'	5' ACTCCAGAAGACCAGAGGAAAT 3'
MCP-1	5' AGCACCAGCCAACTCTCAC 3'	5' TCTGGACCCATTCCTTCTTG 3'
miR-377-loop	5' CTCAACTGGTGTCGTGGAGTCGGCAAT TCAGTTGAGACAAAAGT 3'	
miR-377	5' GTCGTGGAGTCGGCAATT 3'	5' GGCATCACACAAAGGCAAC 3'
miR-145-loop	5' CTCAACTGGTGTCGTGGAGTCGGCAATT CAGTTGAGAGGGATTC 3'	
miR-145	5' CTGGTAGGGTCCAGTTTTC 3'	5' TCAACTGGTGTCGTGGAG 3'
miR-103-loop	5' CTCAACTGGTGTCGTGGAGTCGGCA ATTCAGTTGAGTCATAGCCC 3'	
miR-103	5' CTGGTAGGAGCAGCATTGTA 3'	5' TCAACTGGTGTCGTGGAG 3'
u6-loop	5' CTCAACTGGTGTCGTGGAGTCGGCAATTCA GTTGAGAAAAATATGGAACGCT 3'	
u6	5' CTGGTAGGGTGCTCGCTTCGGCAG 3'	5' CAACTGGTGTCGTGGAGTCGGC 3'
